# Bis­(2-bromo­eth­yl)ammonium bromide

**DOI:** 10.1107/S1600536812033417

**Published:** 2012-07-28

**Authors:** Kamentheren Padayachy, Manuel A. Fernandes, Helder M. Marques, Alvaro S. de Sousa

**Affiliations:** a School of Chemistry, Molecular Sciences Institute, University of the Witwatersrand, Private Bag 3, Wits 2050, Johannesburg, South Africa

## Abstract

The title salt, C_4_H_10_Br_2_N^+^·Br^−^, crystallizes with four cations and four anions in the asymmetric unit. In the crystal, the bis­(2-bromo­eth­yl)ammonium cations and bromide anions are linked into chains by N—H⋯Br hydrogen bonds describing a binary *C*_2_^1^(4) motif along [010]. Each of these chains is formed by a unique cation and anion pair. The ammonium cations occur in the less preferred *anti* conformation, characterized by different NCCBr torsion angles. Adjacent chains are linked by weak C—H⋯Br inter­actions, forming a three-dimensional network. The crystal studied was a pseudo-merohedral twin with twin ratio 0.640 (2):0.360 (2).

## Related literature

For structures of related 2-haloethyl­ammonium salts, see: Bojan *et al.* (2008 ▶); Briggs *et al.* (2004 ▶); Fischer *et al.* (1994 ▶); Kane *et al.* (1992 ▶); Kumar *et al.* (1998 ▶). For graph-set analysis, see: Bernstein *et al.* (1995 ▶). For the preparation of *N*-bis­(2-bromo­ethyl­amine) hydro­bromide, see: Pettit *et al.* (1964) ▶. 
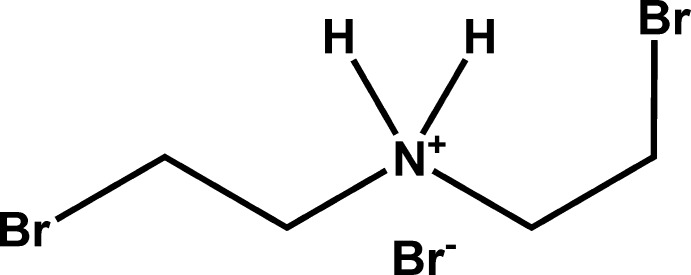


## Experimental

### 

#### Crystal data

C_4_H_10_Br_2_N^+^·Br^−^
*M* *_r_* = 311.86
Monoclinic, 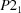

*a* = 15.8861 (13) Å
*b* = 7.4891 (6) Å
*c* = 17.1018 (18) Å
β = 117.450 (5)°
*V* = 1805.6 (3) Å^3^
*Z* = 8
Mo *K*α radiation
μ = 13.32 mm^−1^
*T* = 173 K
0.59 × 0.08 × 0.02 mm

#### Data collection

Bruker APEXII CCD area-detector diffractometer
Absorption correction: integration [face indexed absorption corrections carried out with *XPREP* (Bruker, 2005 ▶)] *T*_min_ = 0.083, *T*_max_ = 0.552
10233 measured reflections
6819 independent reflections
4058 reflections with *I* > 2σ(*I*)
*R*_int_ = 0.143

#### Refinement

*R*[*F* ^2^ > 2σ(*F* ^2^)] = 0.107
*wR*(*F* ^2^) = 0.285
*S* = 0.98
6819 reflections
290 parameters
85 restraints
H-atom parameters constrained
Δρ_max_ = 2.13 e Å^−3^
Δρ_min_ = −2.01 e Å^−3^
Absolute structure: Flack (1983 ▶), 2573 Friedel pairs
Flack parameter: 0.15 (12)

### 

Data collection: *APEX2* (Bruker, 2005 ▶); cell refinement: *APEX2* (Bruker, 2005 ▶); data reduction: *SAINT-NT* (Bruker, 2005 ▶); program(s) used to solve structure: *SHELXS97* (Sheldrick, 2008 ▶); program(s) used to refine structure: *SHELXL97* (Sheldrick, 2008 ▶); molecular graphics: *PLATON* (Spek, 2009 ▶); software used to prepare material for publication: *WinGX* (Farrugia, 1999 ▶) and *PLATON*.

## Supplementary Material

Crystal structure: contains datablock(s) global, I. DOI: 10.1107/S1600536812033417/lr2074sup1.cif

Structure factors: contains datablock(s) I. DOI: 10.1107/S1600536812033417/lr2074Isup2.hkl

Supplementary material file. DOI: 10.1107/S1600536812033417/lr2074Isup3.cml

Additional supplementary materials:  crystallographic information; 3D view; checkCIF report

## Figures and Tables

**Table 1:** Selected torsion angles (°)

Br1—C1—C2—N1	65 (3)
N1—C3—C4—Br2	172 (2)
Br6—C5—C6—N2	176 (2)
N2—C7—C8—Br5	−63 (4)
Br9—C9—C10—N3	−57 (4)
N3—C11—C12—Br8	−169 (2)
Br12—C13—C14—N4	−62 (3)
N4—C15—C16—Br11	−169 (2)

**Table 2:** Hydrogen-bond geometry (Å, °)

*D*—H⋯*A*	*D*—H	H⋯*A*	*D*⋯*A*	*D*—H⋯*A*
N1—H1*A*⋯Br4	0.92	2.34	3.26 (3)	175
N1—H1*B*⋯Br4^i^	0.92	2.41	3.31 (3)	165
N2—H2*B*⋯Br3	0.92	2.30	3.18 (3)	161
N2—H2*A*⋯Br3^ii^	0.92	2.37	3.23 (3)	157
N3—H3*B*⋯Br7	0.92	2.37	3.29 (3)	178
N3—H3*A*⋯Br7^iii^	0.92	2.46	3.33 (3)	159
N4—H4*B*⋯Br10	0.92	2.35	3.27 (3)	178
N4—H4*A*⋯Br10^iv^	0.92	2.40	3.29 (3)	162
C1—H1*D*⋯Br12^v^	1.00	2.92	3.66 (3)	131
C2—H2*C*⋯Br4^vi^	0.99	2.93	3.73 (4)	138
C2—H2*D*⋯Br3	0.99	2.87	3.70 (4)	143
C3—H3*C*⋯Br8	0.98	2.87	3.84 (5)	170
C7—H7*B*⋯Br3^vii^	0.99	2.69	3.68 (5)	173
C9—H9*A*⋯Br1^viii^	0.99	2.87	3.65 (3)	137
C10—H10*A*⋯Br7^vi^	0.99	2.90	3.77 (4)	148
C10—H10*B*⋯Br10^iii^	0.99	2.82	3.72 (4)	153
C12—H12*A*⋯Br2	1.00	2.83	3.73 (5)	150
C14—H14*A*⋯Br10^vi^	0.99	2.93	3.75 (4)	142
C14—H14*B*⋯Br7^iii^	0.99	2.88	3.74 (4)	145
C15—H15*B*⋯Br2	0.99	2.88	3.87 (5)	173
C16—H16*A*⋯Br6^i^	0.99	2.83	3.66 (5)	141

Symmetry codes: (i) 
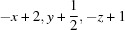
; (ii) 
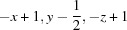
; (iii) 
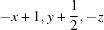
; (iv) 
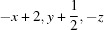
; (v) 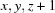; (vi) 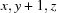; (vii) 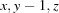; (viii) 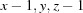.

**Fig. 1.**
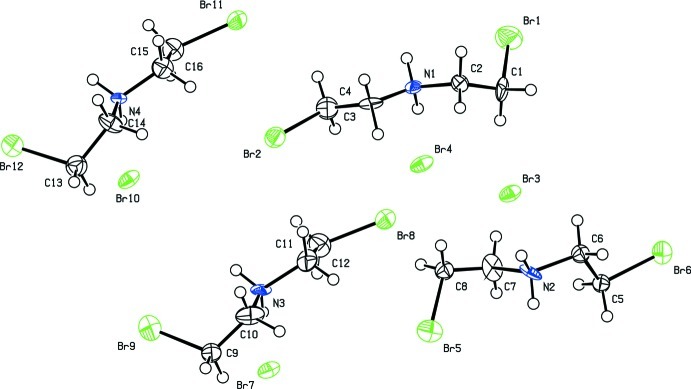
The asymmetric unit of (1). Displacement ellipsoids are drawn at the 50% probability level.

**Fig. 2.**
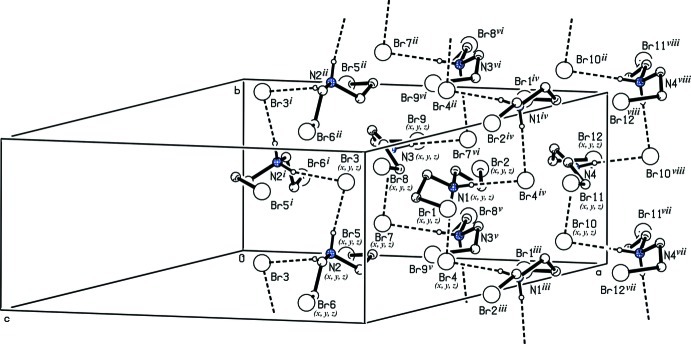
Chains along [010] arising from intermolecular N—H···Br hydrogen bonds. [symmetry codes: (i) -x+1, y+1/2,-z+1; (ii) x,y+1,z; (iii) -x+2, y-1/2,-z+1; (iv) -x+2, y+1/2, -z+1; (v) -x+1,y-1/2,-z; (vi) -x+1, y+1/2,-z; (vii) -x+2,y-1/2, -z; (viii) -x+2, y+1/2,-z]

**Fig. 3.**
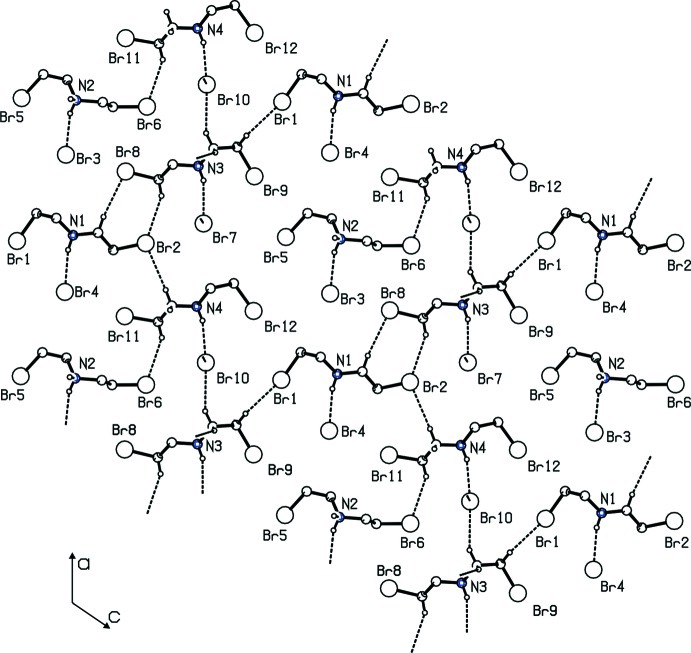
Weak C—H···Br interactions linking chains into a three-dimensional network parallel to (101).

## supplementary crystallographic information

### Comment

Conformational analysis of 2-fluoroethylammonium hydrochloride compounds indicate *gauche* relationships between C—F and C—N bonds are inevitably preferred (Briggs *et al.*, 2004). Stabilization of related *syn* conformers is attributed to weak stereoelectronic gauche effect and/or favourable intramolecular F···H—N^+^ hydrogen bonding interactions. *Syn* conformers exhibiting *gauche* relationships have also been observed in metal complexes (Kumar *et al.*, 1998) and adducts (Kane *et al.*, 1992) of these compounds. However, the *anti* conformation has been observed in the solid state for a N-alkylated derivative (Bojan *et al.*, 2008) and in 2-chloroethylammonium hydrochloride (Fischer *et al.*, 1994). The latter structure shows pairs of *anti* bis(2-chloroethyl)ammonium cations are linked *via* N—H···Cl hydrogen bonds to a *syn* cation to form chains along (1 0 0). In the solid state structure of the title compound (I), only *anti* conformations of bis(2-bromoethyl) ammonium cations (Figure 1) are observed, that are characterised by different NCCBr torsion angles (Table 1). Discreet intermolecular N—H···Br hydrogen bonding interactions (Table 2) mimic N-H···Cl interactions of 2-haloethylammonium compounds (Briggs *et al.*, 2004, Fischer *et al.*, 1994), and link cations into staggered chains along the *b*-axis, to define a binary *C*_2_^1^(4) motif (Bernstein *et al.*, 1995). Weak van der Waals C—H···Br interactions link 2-bromoethylammonium cations into layers parallel to the *ac* plane. The structure is also stabilized by several Br···Br interactions, the shortest being between Br3 and Br8 [3.559 (4) Å], and between Br2 and Br7 [3.594 (5) Å].

### Experimental

N-Bis(2-bromoethylamine) hydrobromide was prepared as reported by Pettit *et al.* (1964). Diethanolamine (12 g, 0.114 mol) was added, with cooling, to 100 mL of 48% HBr. The reaction vessel was fitted with a Vigreaux column and Dean-Stark apparatus and the solution heated collecting approximately 70 mL of water through azeotropic distillation. The remaining HBr was removed under reduced pressure to yield viscous orange oil that crystallized upon cooling.

^1^H(D_2_O, 300 MHz) 3.342 (4*H*, t, CH_2_NH), 3.992 (4*H*, t,CH_2_Br).

### Refinement

Crystals of the title compound seem to be inherently pseudo-merohedrally twinned as several recrystallizations led to twinned crystals. The pseudo-merohedral twin is approximately described by the twin law [-1.00 0.00 0.00 0.00 -1.00 0.00 1.00 0.00 1.00]. Hydrogen atoms were visible in the difference map and those bonded to carbon atoms were positioned geometrically and allowed for as riding atoms with C—H = 0.99 Å (CH_2_) and N—H = 0.92 Å (NH_2_). The coordinates of hydrogen atoms involved in hydrogen bonding were refined freely. During the refinements the *U_iso_*(H) values were set at 1.2*U_eq_* of the parent atom.

### Crystal data

**Table:**

C_4_H_10_Br_2_N^+^·Br^−^	*F*(000) = 1168
*M**_r_* = 311.86	*D*_x_ = 2.294 Mg m^−^^3^
Monoclinic, *P*2_1_	Mo *K*α radiation, λ = 0.71073 Å
Hall symbol: P 2yb	Cell parameters from 1740 reflections
*a* = 15.8861 (13) Å	θ = 2.7–28.0°
*b* = 7.4891 (6) Å	µ = 13.32 mm^−^^1^
*c* = 17.1018 (18) Å	*T* = 173 K
β = 117.450 (5)°	Needle, colourless
*V* = 1805.6 (3) Å^3^	0.59 × 0.08 × 0.02 mm
*Z* = 8	

### Data collection

**Table:**

Bruker APEXII CCD area-detector diffractometer	6819 independent reflections
Radiation source: fine-focus sealed tube	4058 reflections with *I* > 2σ(*I*)
Graphite monochromator	*R*_int_ = 0.143
phi and ω scans	θ_max_ = 27.0°, θ_min_ = 1.3°
Absorption correction: integration [face indexed absorption corrections carried out with *XPREP* (Bruker, 2005)]	*h* = −19→20
*T*_min_ = 0.083, *T*_max_ = 0.552	*k* = −9→9
10233 measured reflections	*l* = −21→8

### Refinement

**Table:**

Refinement on *F*^2^	Secondary atom site location: difference Fourier map
Least-squares matrix: full	Hydrogen site location: inferred from neighbouring sites
*R*[*F*^2^ > 2σ(*F*^2^)] = 0.107	H-atom parameters constrained
*wR*(*F*^2^) = 0.285	*w* = 1/[σ^2^(*F*_o_^2^) + (0.1648*P*)^2^] where *P* = (*F*_o_^2^ + 2*F*_c_^2^)/3
*S* = 0.98	(Δ/σ)_max_ < 0.001
6819 reflections	Δρ_max_ = 2.13 e Å^−^^3^
290 parameters	Δρ_min_ = −2.01 e Å^−^^3^
85 restraints	Absolute structure: Flack (1983), 2573 Friedel pairs
Primary atom site location: structure-invariant direct methods	Flack parameter: 0.15 (12)

### Special details

**Table:**

Geometry. All esds (except the esd in the dihedral angle between two l.s. planes) are estimated using the full covariance matrix. The cell esds are taken into account individually in the estimation of esds in distances, angles and torsion angles; correlations between esds in cell parameters are only used when they are defined by crystal symmetry. An approximate (isotropic) treatment of cell esds is used for estimating esds involving l.s. planes.
Refinement. Refinement of F^2^ against ALL reflections. The weighted R-factor wR and goodness of fit S are based on F^2^, conventional R-factors R are based on F, with F set to zero for negative F^2^. The threshold expression of F^2^ > 2sigma(F^2^) is used only for calculating R-factors(gt) etc. and is not relevant to the choice of reflections for refinement. R-factors based on F^2^ are statistically about twice as large as those based on F, and R- factors based on ALL data will be even larger.

### Fractional atomic coordinates and isotropic or equivalent isotropic displacement parameters (Å^2^)

**Table:**

	*x*	*y*	*z*	*U*_iso_*/*U*_eq_	
C1	0.890 (2)	0.595 (5)	0.623 (2)	0.032 (8)	
H1C	0.8598	0.4761	0.6060	0.038*	
H1D	0.8621	0.6552	0.6580	0.038*	
C2	0.868 (2)	0.699 (5)	0.5452 (19)	0.023 (7)	
H2C	0.9002	0.8161	0.5628	0.028*	
H2D	0.7989	0.7205	0.5133	0.028*	
C3	0.842 (3)	0.666 (5)	0.388 (2)	0.032 (8)	
H3C	0.7739	0.6447	0.3672	0.038*	
H3D	0.8522	0.7949	0.3824	0.038*	
C4	0.877 (3)	0.559 (5)	0.334 (2)	0.038 (10)	
H4C	0.8756	0.4302	0.3461	0.046*	
H4D	0.9425	0.5935	0.3487	0.046*	
N1	0.8992 (19)	0.607 (4)	0.4843 (16)	0.024 (6)	
H1A	0.8926	0.4855	0.4877	0.029*	
H1B	0.9624	0.6305	0.5030	0.029*	
Br1	1.0262 (4)	0.5657 (7)	0.6975 (3)	0.0567 (14)	
Br2	0.7909 (3)	0.6111 (6)	0.2100 (2)	0.0343 (9)	
C5	0.635 (2)	0.016 (4)	0.6613 (18)	0.024 (7)	
H5A	0.6675	−0.0672	0.6393	0.028*	
H5B	0.5687	−0.0248	0.6399	0.028*	
C6	0.636 (2)	0.202 (4)	0.6290 (19)	0.024 (7)	
H6A	0.7028	0.2446	0.6549	0.029*	
H6B	0.6010	0.2825	0.6493	0.029*	
C7	0.649 (3)	0.109 (6)	0.492 (2)	0.041 (9)	
H7A	0.7174	0.1302	0.5296	0.049*	
H7B	0.6366	−0.0201	0.4924	0.049*	
C8	0.623 (2)	0.165 (4)	0.4031 (18)	0.027 (7)	
H8A	0.6652	0.1042	0.3834	0.033*	
H8B	0.6350	0.2953	0.4036	0.033*	
N2	0.5926 (19)	0.211 (4)	0.5289 (15)	0.027 (7)	
H2A	0.5321	0.1649	0.5050	0.032*	
H2B	0.5880	0.3284	0.5119	0.032*	
Br5	0.4962 (3)	0.1204 (6)	0.3201 (2)	0.0408 (9)	
Br6	0.7005 (3)	0.0235 (6)	0.7903 (2)	0.0346 (9)	
C9	0.273 (2)	0.611 (5)	−0.1351 (19)	0.029 (7)	
H9A	0.2123	0.6703	−0.1729	0.034*	
H9B	0.2592	0.4882	−0.1235	0.034*	
C10	0.321 (2)	0.710 (5)	−0.048 (2)	0.039 (9)	
H10A	0.3297	0.8364	−0.0594	0.047*	
H10B	0.2788	0.7064	−0.0197	0.047*	
C11	0.455 (3)	0.690 (5)	0.106 (2)	0.034 (9)	
H11A	0.4765	0.8159	0.1101	0.040*	
H11B	0.4065	0.6839	0.1261	0.040*	
C12	0.538 (3)	0.574 (6)	0.163 (2)	0.046 (11)	
H12A	0.5926	0.5979	0.1510	0.055*	
H12B	0.5207	0.4463	0.1507	0.055*	
N3	0.4142 (18)	0.629 (4)	0.0114 (15)	0.023 (6)	
H3A	0.4564	0.6549	−0.0098	0.028*	
H3B	0.4074	0.5064	0.0101	0.028*	
Br8	0.5708 (3)	0.6336 (6)	0.2858 (2)	0.0334 (9)	
Br9	0.3459 (3)	0.5968 (8)	−0.1998 (3)	0.0546 (13)	
C13	0.769 (3)	0.542 (5)	−0.121 (2)	0.037 (9)	
H13A	0.7071	0.6012	−0.1541	0.045*	
H13B	0.7583	0.4202	−0.1056	0.045*	
C14	0.830 (2)	0.647 (5)	−0.037 (2)	0.031 (8)	
H14A	0.8405	0.7689	−0.0532	0.037*	
H14B	0.7944	0.6584	−0.0024	0.037*	
C15	0.966 (3)	0.625 (5)	0.111 (2)	0.035 (8)	
H15A	0.9812	0.7533	0.1129	0.042*	
H15B	0.9195	0.6104	0.1342	0.042*	
C16	1.051 (3)	0.525 (5)	0.165 (2)	0.038 (9)	
H16A	1.1015	0.5575	0.1491	0.046*	
H16B	1.0382	0.3959	0.1546	0.046*	
N4	0.9227 (19)	0.561 (4)	0.0182 (16)	0.026 (6)	
H4A	0.9634	0.5856	−0.0051	0.031*	
H4B	0.9147	0.4394	0.0172	0.031*	
Br11	1.0931 (3)	0.5783 (5)	0.2892 (2)	0.0327 (10)	
Br12	0.8319 (3)	0.5275 (7)	−0.1957 (3)	0.0483 (11)	
Br3	0.6231 (2)	0.6258 (6)	0.5118 (2)	0.0284 (8)	
Br4	0.8830 (2)	0.1738 (4)	0.4877 (2)	0.0282 (9)	
Br7	0.3904 (2)	0.1919 (5)	0.0112 (2)	0.0289 (9)	
Br10	0.8945 (2)	0.1288 (5)	0.0195 (2)	0.0284 (8)	

### Atomic displacement parameters (Å^2^)

**Table:**

	*U*^11^	*U*^22^	*U*^33^	*U*^12^	*U*^13^	*U*^23^
C1	0.033 (9)	0.032 (9)	0.032 (9)	0.000 (5)	0.017 (6)	−0.001 (5)
C2	0.024 (8)	0.021 (8)	0.023 (8)	0.003 (5)	0.009 (5)	−0.002 (5)
C3	0.030 (9)	0.033 (10)	0.033 (9)	0.000 (5)	0.015 (6)	0.000 (5)
C4	0.039 (11)	0.038 (11)	0.036 (10)	0.000 (5)	0.017 (6)	0.000 (5)
N1	0.032 (15)	0.014 (13)	0.034 (13)	−0.009 (12)	0.022 (13)	−0.002 (13)
Br1	0.042 (3)	0.070 (4)	0.044 (2)	−0.009 (2)	0.008 (2)	0.005 (2)
Br2	0.0287 (19)	0.041 (2)	0.0319 (18)	0.000 (2)	0.0127 (16)	0.005 (2)
C5	0.025 (8)	0.026 (8)	0.022 (8)	0.000 (5)	0.013 (5)	0.001 (5)
C6	0.024 (8)	0.025 (8)	0.026 (8)	0.001 (5)	0.013 (5)	0.001 (5)
C7	0.042 (10)	0.040 (10)	0.041 (10)	0.001 (5)	0.020 (6)	−0.002 (5)
C8	0.025 (8)	0.029 (9)	0.028 (8)	−0.001 (5)	0.013 (5)	0.000 (5)
N2	0.032 (17)	0.012 (12)	0.019 (12)	−0.006 (12)	−0.003 (12)	−0.003 (11)
Br5	0.034 (2)	0.037 (2)	0.049 (2)	0.004 (2)	0.0167 (18)	−0.005 (2)
Br6	0.032 (2)	0.040 (2)	0.0327 (19)	0.0076 (17)	0.0154 (17)	0.0036 (17)
C9	0.026 (8)	0.027 (8)	0.031 (8)	−0.001 (5)	0.011 (5)	−0.001 (5)
C10	0.012 (17)	0.04 (2)	0.06 (2)	0.003 (16)	0.008 (17)	−0.005 (19)
C11	0.05 (3)	0.017 (15)	0.05 (2)	−0.006 (16)	0.04 (2)	0.001 (16)
C12	0.045 (11)	0.047 (12)	0.045 (11)	0.002 (5)	0.022 (7)	−0.002 (5)
N3	0.023 (7)	0.022 (7)	0.024 (7)	−0.001 (5)	0.011 (5)	−0.002 (5)
Br8	0.0307 (19)	0.037 (2)	0.0353 (18)	−0.0017 (19)	0.0178 (16)	−0.0001 (19)
Br9	0.055 (3)	0.070 (3)	0.039 (2)	−0.010 (3)	0.022 (2)	−0.006 (2)
C13	0.037 (10)	0.037 (10)	0.037 (10)	0.000 (5)	0.016 (6)	0.003 (5)
C14	0.027 (18)	0.022 (18)	0.032 (17)	0.002 (15)	0.003 (15)	−0.011 (15)
C15	0.034 (9)	0.034 (9)	0.035 (9)	0.003 (5)	0.014 (6)	−0.002 (5)
C16	0.036 (10)	0.038 (10)	0.038 (10)	0.004 (5)	0.016 (6)	−0.003 (5)
N4	0.023 (14)	0.032 (16)	0.019 (12)	−0.017 (12)	0.007 (12)	−0.005 (11)
Br11	0.026 (2)	0.041 (2)	0.0324 (19)	−0.0030 (17)	0.0152 (17)	0.0005 (16)
Br12	0.054 (3)	0.054 (3)	0.041 (2)	−0.018 (2)	0.025 (2)	−0.009 (2)
Br3	0.0292 (18)	0.0185 (18)	0.0437 (18)	−0.0031 (16)	0.0221 (15)	−0.0011 (17)
Br4	0.0198 (17)	0.023 (2)	0.047 (2)	0.0011 (13)	0.0193 (16)	0.0033 (15)
Br7	0.0259 (19)	0.0210 (19)	0.046 (2)	−0.0032 (14)	0.0216 (17)	−0.0019 (15)
Br10	0.0318 (19)	0.0171 (17)	0.0480 (19)	−0.0017 (16)	0.0284 (17)	0.0005 (17)

### Geometric parameters (Å, º)

**Table:**

C1—C2	1.44 (5)	C9—C10	1.51 (5)
C1—Br1	1.96 (3)	C9—Br9	1.93 (3)
C1—H1C	0.9900	C9—H9A	0.9900
C1—H1D	0.9900	C9—H9B	0.9900
C2—N1	1.51 (4)	C10—N3	1.49 (4)
C2—H2C	0.9900	C10—H10A	0.9900
C2—H2D	0.9900	C10—H10B	0.9900
C3—C4	1.50 (5)	C11—C12	1.50 (5)
C3—N1	1.53 (4)	C11—N3	1.51 (4)
C3—H3C	0.9900	C11—H11A	0.9900
C3—H3D	0.9900	C11—H11B	0.9900
C4—Br2	1.96 (3)	C12—Br8	1.97 (4)
C4—H4C	0.9900	C12—H12A	0.9900
C4—H4D	0.9900	C12—H12B	0.9900
N1—H1A	0.9200	N3—H3A	0.9200
N1—H1B	0.9200	N3—H3B	0.9200
C5—C6	1.50 (4)	C13—C14	1.53 (5)
C5—Br6	1.96 (3)	C13—Br12	1.94 (4)
C5—H5A	0.9900	C13—H13A	0.9900
C5—H5B	0.9900	C13—H13B	0.9900
C6—N2	1.52 (4)	C14—N4	1.48 (4)
C6—H6A	0.9900	C14—H14A	0.9900
C6—H6B	0.9900	C14—H14B	0.9900
C7—C8	1.44 (5)	C15—C16	1.45 (5)
C7—N2	1.51 (5)	C15—N4	1.49 (4)
C7—H7A	0.9900	C15—H15A	0.9900
C7—H7B	0.9900	C15—H15B	0.9900
C8—Br5	1.89 (3)	C16—Br11	1.95 (3)
C8—H8A	0.9900	C16—H16A	0.9900
C8—H8B	0.9900	C16—H16B	0.9900
N2—H2A	0.9200	N4—H4A	0.9200
N2—H2B	0.9200	N4—H4B	0.9200
			
C2—C1—Br1	112 (2)	C10—C9—Br9	116 (2)
C2—C1—H1C	109.2	C10—C9—H9A	108.4
Br1—C1—H1C	109.2	Br9—C9—H9A	108.4
C2—C1—H1D	109.2	C10—C9—H9B	108.4
Br1—C1—H1D	109.2	Br9—C9—H9B	108.4
H1C—C1—H1D	107.9	H9A—C9—H9B	107.4
C1—C2—N1	112 (3)	N3—C10—C9	111 (3)
C1—C2—H2C	109.1	N3—C10—H10A	109.4
N1—C2—H2C	109.1	C9—C10—H10A	109.4
C1—C2—H2D	109.1	N3—C10—H10B	109.4
N1—C2—H2D	109.1	C9—C10—H10B	109.4
H2C—C2—H2D	107.8	H10A—C10—H10B	108.0
C4—C3—N1	108 (3)	C12—C11—N3	110 (3)
C4—C3—H3C	110.1	C12—C11—H11A	109.8
N1—C3—H3C	110.1	N3—C11—H11A	109.8
C4—C3—H3D	110.1	C12—C11—H11B	109.8
N1—C3—H3D	110.1	N3—C11—H11B	109.8
H3C—C3—H3D	108.4	H11A—C11—H11B	108.2
C3—C4—Br2	107 (2)	C11—C12—Br8	106 (3)
C3—C4—H4C	110.3	C11—C12—H12A	110.5
Br2—C4—H4C	110.3	Br8—C12—H12A	110.5
C3—C4—H4D	110.3	C11—C12—H12B	110.5
Br2—C4—H4D	110.3	Br8—C12—H12B	110.5
H4C—C4—H4D	108.6	H12A—C12—H12B	108.6
C2—N1—C3	113 (3)	C10—N3—C11	114 (3)
C2—N1—H1A	108.9	C10—N3—H3A	108.7
C3—N1—H1A	108.9	C11—N3—H3A	108.7
C2—N1—H1B	108.9	C10—N3—H3B	108.7
C3—N1—H1B	108.9	C11—N3—H3B	108.7
H1A—N1—H1B	107.8	H3A—N3—H3B	107.6
C6—C5—Br6	107 (2)	C14—C13—Br12	111 (3)
C6—C5—H5A	110.2	C14—C13—H13A	109.4
Br6—C5—H5A	110.2	Br12—C13—H13A	109.4
C6—C5—H5B	110.2	C14—C13—H13B	109.4
Br6—C5—H5B	110.2	Br12—C13—H13B	109.4
H5A—C5—H5B	108.5	H13A—C13—H13B	108.0
C5—C6—N2	112 (3)	N4—C14—C13	113 (3)
C5—C6—H6A	109.2	N4—C14—H14A	109.1
N2—C6—H6A	109.2	C13—C14—H14A	109.1
C5—C6—H6B	109.2	N4—C14—H14B	109.1
N2—C6—H6B	109.2	C13—C14—H14B	109.1
H6A—C6—H6B	107.9	H14A—C14—H14B	107.8
C8—C7—N2	111 (3)	C16—C15—N4	111 (3)
C8—C7—H7A	109.4	C16—C15—H15A	109.5
N2—C7—H7A	109.4	N4—C15—H15A	109.5
C8—C7—H7B	109.4	C16—C15—H15B	109.5
N2—C7—H7B	109.4	N4—C15—H15B	109.5
H7A—C7—H7B	108.0	H15A—C15—H15B	108.1
C7—C8—Br5	115 (2)	C15—C16—Br11	110 (3)
C7—C8—H8A	108.5	C15—C16—H16A	109.6
Br5—C8—H8A	108.5	Br11—C16—H16A	109.6
C7—C8—H8B	108.5	C15—C16—H16B	109.6
Br5—C8—H8B	108.5	Br11—C16—H16B	109.6
H8A—C8—H8B	107.5	H16A—C16—H16B	108.1
C7—N2—C6	113 (3)	C14—N4—C15	112 (3)
C7—N2—H2A	108.9	C14—N4—H4A	109.2
C6—N2—H2A	108.9	C15—N4—H4A	109.2
C7—N2—H2B	108.9	C14—N4—H4B	109.2
C6—N2—H2B	108.9	C15—N4—H4B	109.2
H2A—N2—H2B	107.7	H4A—N4—H4B	107.9
			
Br1—C1—C2—N1	65 (3)	Br9—C9—C10—N3	−57 (4)
N1—C3—C4—Br2	172 (2)	N3—C11—C12—Br8	−169 (2)
C1—C2—N1—C3	155 (3)	C9—C10—N3—C11	−167 (3)
C4—C3—N1—C2	−177 (3)	C12—C11—N3—C10	168 (3)
Br6—C5—C6—N2	176 (2)	Br12—C13—C14—N4	−62 (3)
N2—C7—C8—Br5	−63 (4)	N4—C15—C16—Br11	−169 (2)
C8—C7—N2—C6	−162 (3)	C13—C14—N4—C15	−160 (3)
C5—C6—N2—C7	−66 (4)	C16—C15—N4—C14	174 (3)

### Hydrogen-bond geometry (Å, º)

**Table:**

*D*—H···*A*	*D*—H	H···*A*	*D*···*A*	*D*—H···*A*
N1—H1*A*···Br4	0.92	2.34	3.26 (3)	175
N1—H1*B*···Br4^i^	0.92	2.41	3.31 (3)	165
N2—H2*B*···Br3	0.92	2.30	3.18 (3)	161
N2—H2*A*···Br3^ii^	0.92	2.37	3.23 (3)	157
N3—H3*B*···Br7	0.92	2.37	3.29 (3)	178
N3—H3*A*···Br7^iii^	0.92	2.46	3.33 (3)	159
N4—H4*B*···Br10	0.92	2.35	3.27 (3)	178
N4—H4*A*···Br10^iv^	0.92	2.40	3.29 (3)	162
C1—H1*D*···Br12^v^	1.00	2.92	3.66 (3)	131
C2—H2*C*···Br4^vi^	0.99	2.93	3.73 (4)	138
C2—H2*D*···Br3	0.99	2.87	3.70 (4)	143
C3—H3*C*···Br8	0.98	2.87	3.84 (5)	170
C7—H7*B*···Br3^vii^	0.99	2.69	3.68 (5)	173
C9—H9*A*···Br1^viii^	0.99	2.87	3.65 (3)	137
C10—H10*A*···Br7^vi^	0.99	2.90	3.77 (4)	148
C10—H10*B*···Br10^iii^	0.99	2.82	3.72 (4)	153
C12—H12*A*···Br2	1.00	2.83	3.73 (5)	150
C14—H14*A*···Br10^vi^	0.99	2.93	3.75 (4)	142
C14—H14*B*···Br7^iii^	0.99	2.88	3.74 (4)	145
C15—H15*B*···Br2	0.99	2.88	3.87 (5)	173
C16—H16*A*···Br6^i^	0.99	2.83	3.66 (5)	141

Symmetry codes: (i) −*x*+2, *y*+1/2, −*z*+1; (ii) −*x*+1, *y*−1/2, −*z*+1; (iii) −*x*+1, *y*+1/2, −*z*; (iv) −*x*+2, *y*+1/2, −*z*; (v) *x*, *y*, *z*+1; (vi) *x*, *y*+1, *z*; (vii) *x*, *y*−1, *z*; (viii) *x*−1, *y*, *z*−1.

## References

[bb1] Bernstein, J., Davis, R. E., Shinoni, L. & Chang, N.-L. (1995). *Angew. Chem. Int. Ed. Engl.* **34**, 1555–1573.

[bb2] Bojan, R. V., Varga, R. A. & Silvestru, C. (2008). *Acta Cryst.* E**64**, o86. 10.1107/S1600536807056279 PMC2915042 21200963

[bb3] Briggs, C. R. S., Allen, M. J., O’Hagan, D., Tozer, D. J., Slawin, A. M. Z., Goeta, A. E. & Howard, J. A. K. (2004). *Org. Biomol. Chem.* **2**, 732–740. 10.1039/b312188g 14985814

[bb4] Bruker (2005). *APEX2* and *SAINT-NT* and *XPREP* Bruker AXS Inc., Madison, Wisconsin, USA.

[bb5] Farrugia, L. J. (1999). *J. Appl. Cryst.* **32**, 837–838.

[bb6] Fischer, A., Neda, I., Jones, P. G. & Schmutzler, R. (1994). *Phosphorus Sulfur Silicon Relat. Elem.* **91**, 103–127.

[bb7] Flack, H. D. (1983). *Acta Cryst.* A**39**, 876–881.

[bb8] Kane, C. J., Long, R., Pettit, W. E., Breneman, G. L. & Pettit, G. R. (1992). *Acta Cryst.* C**48**, 1490–1491. 10.1107/s0108270191015135 1418816

[bb9] Kumar, J. S., Singh, A. K., Yang, J. & Drake, J. E. (1998). *J. Coord. Chem.* **44**, 217–223.

[bb12] Pettit, G. R., Chamberland, M. R., Blonda, D. S. & Vickers, M. A. (1964). *Can. J. Chem.* **42**, 1699–1706.

[bb10] Sheldrick, G. M. (2008). *Acta Cryst.* A**64**, 112–122. 10.1107/S0108767307043930 18156677

[bb11] Spek, A. L. (2009). *Acta Cryst.* D**65**, 148–155. 10.1107/S090744490804362X PMC2631630 19171970

